# Sex as a Potential Moderator for Baclofen Response in the Treatment of Alcohol Dependence

**DOI:** 10.3389/fgwh.2022.807269

**Published:** 2022-03-29

**Authors:** Kirsten C. Morley, Eva Louie, Tristan Hurzeler, Andrew Baillie, Glenys Dore, Nghi Phung, Paul S. Haber

**Affiliations:** ^1^Faculty of Medicine and Health, Central Clinical School, Specialty of Addiction Medicine, Sydney Medical School, University of Sydney, Darlington, NSW, Australia; ^2^Edith Collins Centre, Royal Prince Alfred Hospital, Sydney Local Health District, Sydney, NSW, Australia; ^3^Faculty of Medicine and Health, School of Health Sciences, University of Sydney, Darlington, NSW, Australia; ^4^Herbert St Alcohol Clinic, Royal North Shore Hospital, Sydney, NSW, Australia; ^5^Centre for Addiction Medicine, Westmead Hospital, Parramatta, NSW, Australia

**Keywords:** sex, baclofen, alcohol dependence, alcohol use disorder, gender, treatment, pharmacotherapy

## Abstract

**Background and Aims:**

Recent studies indicate that sex may moderate the response to baclofen in the treatment of alcohol use disorder (AUD). We conducted a secondary analysis of a double-blind randomized controlled trial, Baclofen in the treatment of Alcohol Liver Disease (BacALD), to examine the moderating role of sex on treatment response to baclofen in reducing alcohol consumption.

**Methods:**

Alcohol-dependent patients (*n* = 104 including 74 men and 30 women) were treated for 12 weeks with baclofen (30 mg/day or 75 mg) or placebo. Predefined primary outcomes included time to lapse (any drinking) and relapse (≥ 5 drinks per day in men and ≥ 4drinks per day in women). Other outcomes included drinks per drinking day, the number of heavy drinking days, and percentage of days abstinent. We also examined the frequency of adverse events with an exploratory dose–response analysis.

**Results:**

There was a main effect of baclofen for days to first lapse for women (Log Rank: χ^2^ = 6.23, *p* = 0.01, *d* = 0.49) but not for men (Log Rank: χ^2^ = 2.48, *p* = 0.12, *d* = 0.22) and a marginal effect of baclofen for days to first relapse for women (Log Rank: χ^2^ = 3.15, *p* = 0.08, *d* = 0.27) but not for men (Log Rank: χ^2^ = 2.03, *p* = 0.16, *d* = 0.17). There were no significant effects of sex on the frequency of adverse events reported for the combined-dose or between-dose analysis (all *p* > 0.44).

**Conclusion:**

Baclofen significantly delayed the time to lapse for women but not male participants. These findings provide some support for the hypothesis that sex may be a potential moderator of baclofen response in the treatment of AUD.

**Trial Registration:**

https://clinicaltrials.gov/ct2/show/NCT01711125, identifier: NCT01711125.

## Introduction

Harmful use of alcohol is responsible for 5.9% of all deaths world-wide and is a causal factor in more than 200 disease and injury conditions (1). Alcohol use disorder (AUD) is a common disorder most often characterized by compulsive alcohol-seeking and consumption despite negative consequences (2). The prevalence of AUD has historically been lowered in women relative to men; however, the rates of AUDs in women are rapidly increasing (3) such that this gap appears to be closing (4).

Previous research has highlighted potential sex differences in the effects of alcohol and also with regard to the patterns of alcohol consumption. The pharmacokinetics of alcohol differs in women tending toward higher blood alcohol concentration (BAC) relative to men (5), which may be due to sex differences in body-water volume and first-pass metabolism (6). Sex hormones also reciprocally interact with alcohol use (7). Some studies have observed elevated BACs and increased behavioral sensitivity to alcohol during mid-luteal and ovulatory phases, as compared with follicular phases (8–10) although there are mixed results with regard to any association between the menstrual cycle phase and alcohol consumption (11). In terms of patterns of alcohol consumption, research suggests that women are generally more likely to drink to regulate stress reactivity and negative affect [for review see Peltier et al. (12)], are more likely to relapse in response to stress, and also more likely to show a sensitized stress response during alcohol withdrawal relative to men (13, 14). Thus, these biological factors may contribute not only to the development of AUD but also to treatment response and recovery.

Unfortunately, however, the consideration of sex differences in clinical research in alcohol treatment response and recovery has been limited. Pharmacotherapy is one of the frontline tools for the management of AUD and to facilitate the reduction of alcohol consumption in both men and women (15). Although existing research generally suggests no major sex differences in terms of overall outcomes in pharmacological treatments such as acamprosate or naltrexone (16, 17), there have only been a small number of studies that have directly tested sex differences and often there is a low rate of women enrolled in clinical trials. Indeed, as recently noted by Schick et al. (18), given a strong rationale to systematically consider sex differences in alcohol research and despite national and international guidelines, a very few articles have conducted subgroup analyses to examine differences in alcohol pharmacotherapy outcomes by sex. These authors emphasize that considerably greater efforts regarding the inclusion, analysis, and reporting of data focused on women are required.

Baclofen, a selective g-aminobutyric acid type B (GABAB) receptor agonist, provides a potential treatment for alcohol dependence, with several clinical trials (19–22), and meta-analyses (23, 24) demonstrating efficacy. We have previously reported that baclofen delays time to lapse and relapse relative to placebo (25) in addition to attenuating alcohol cue-elicited brain activation (26), cardiovascular response (27), and emotional regulation during high threat stimuli (28). Nonetheless, it appears that not all patients respond favorably to baclofen (2, 29), such that further understanding of the mechanisms of action of the pharmacotherapy is still required to facilitate a personalized approach (30). The response to baclofen has previously been found to be predicted by baseline anxiety levels (19) and the level of alcohol consumption (23, 31). Interestingly, one recently completed trial by Garbutt et al. (22) supports a therapeutic effect of baclofen but also indicates the potential of an enhanced treatment effect for women at low doses and reduced tolerability at higher doses compared to men. In this study, women treated with baclofen demonstrated a greater reduction in heavy drinking days and abstinence from alcohol (22).

The current study thus aimed to examine the moderating role of sex on baclofen treatment response (22). We conducted a secondary analysis of Baclofen in the treatment of Alcohol Liver Disease (BacALD) randomized controlled trial results (25) stratified by sex. As per the initial protocol (32) and published results of the main study (25), we *a priori* examined: the primary outcomes time to first lapse (1 drink); time to relapse (≥ 4 drinks for women, ≥ 5 drinks for men); the secondary alcohol consumption that outcomes average drinks per drinking day, the number of heavy drinking days and percentage of days abstinent; and, finally, the frequency of commonly reported adverse events.

## Methods

### Design

The rationale, design, and methods of the main study have been previously detailed (32), and the primary outcomes are reported (25). In brief, after baseline assessment, eligible alcohol-dependent individuals were randomized to placebo, baclofen 30 mg (10 three times a day) and baclofen 75 mg (25 three times a day) for 12 weeks including 74 men (*n* = 23 placebo, *n* = 51 baclofen) and 30 women (*n* = 10 placebo, *n* = 20 baclofen). The study was approved by the Human Ethics Review Committee of the Sydney Local Health District, Northern Sydney Local Health District, and South Western Sydney Local Health District (X11-0154 & X07-0041 & X01-0262), and the main trial was registered in the Clinical Trials Registry (NCT01711125). The study involved off-label use of a registered medication in Australia, and the approval was given under the Clinical Trial Notification (CTN) scheme of the Therapeutics Goods Administration (TGA) (2013/0060).

### Participants and Procedure

Participants were Australian Caucasian men and women who attended an outpatient treatment or follow-up and an inpatient detoxification program or participated in a follow-up or to make a response to advertising. All participants signed informed consent.

Inclusion criteria were: (i) alcohol dependence according to the ICD-10 criteria; (ii) age (18–75); (iii) adequate cognition and English language skills to give a valid consent and complete research interviews; (iv) willingness to give written informed consent; (v) abstinence from alcohol for between 3 and 21 days; (vi) resolution of any clinically evident alcohol withdrawal (CIWA-AR); and (vii) at least 48 h after ceasing any diazepam required for withdrawal management. Exclusion criteria were: (i) active major mental disorder associated with psychosis or a significant suicide risk, (ii) pregnancy or lactation, (iii) concurrent use of any psychotropic medication other than antidepressants (unless taken at stable doses for at least 2 months); (iv) unstable substance use; (v) clinical evidence of persisting hepatic encephalopathy (drowsiness, sleep inversion, or asterixis); (vi) pending incarceration; (vii) the lack of stable housing, (viii) peptic ulcer; and (ix) unstable diabetes mellitus.

### Assessments

A detailed list of assessments has been outlined previously (32). Participants were asked “what is your sex” and their responses were recorded. Briefly, the outcomes for this study were derived from drinking measures in the Time Line Follow Back (TLFB) (33) obtained from structured interviews at baseline and during a 12-week trial period (weeks 1, 3, 6, 9, and 12).

Craving was measured using the Penn Alcohol Craving Scale [PACS (34)]. Anxiety, Depression, and Stress as measured by the Depression Anxiety Stress Scale [DASS (34)]. The frequency of adverse events was recorded as self-reported by participants. Compliance was assessed by self-report, pill count of the returned medication package, the daily monitoring diary, and a urinary analysis of baclofen levels in the randomly selected 50% of participants. Researchers, clinicians, and participants were blinded from treatment allocation.

### Interventions

Participants were allocated 1:1:1 as per a computer-generated randomization sequence provided to the hospital clinical trial pharmacist. Participants in the baclofen 30 mg/day or 75 mg group took a capsule of 10 or 25 mg, respectively: 1 × day for the first 2 days, 2 × day on days 3–4, 3 × day on days 5–80, 2 × day on days 81–82, and finally 1 × day for the last 2 days. The placebo pills, which were identical in appearance, were also titrated upward and downward to maintain the double blind. All participants received 1 medical assessment and 5 follow-up medical reviews over the 12-week treatment period, held at weeks 1, 3, 6, 9, and 12. Participants were medically monitored for adverse events and prescribed the study medication at each appointment. Participants who experienced moderate side effects had their dose reduced according to physician judgement. All participants received a brief compliance therapy, a 4–6 session intervention lasting 20–60 min focussed on enhancing medication compliance (such as targeting ambivalence and misperceptions about medication). Participants were encouraged to defer concurrent psychotherapy until at least week 6 of the trial.

### Outcomes

Primary outcomes were time to first lapse (1 drink) and time to relapse (≥ 4 drinks for women, ≥ 5 drinks for men). Secondary alcohol consumption outcomes included average drinks per drinking day (at week 12 follow-up), the number of heavy drinking days (at week 12 follow-up), and percentage of days abstinent (over the week 12 trial). We also examined the frequency of commonly reported adverse events.

### Statistical Analysis

Analyses were performed on an intention-to-treat basis including all participants who took at least one dose of medication. As previously outlined and published in the initial study protocol (32) and then the main trial results (32), the analyses of primary outcomes included placebo vs. baclofen (composite of the two doses). ANOVA for continuous characteristics and χ^2^ tests for categorical variables were conducted to determine differences between groups at baseline. Survival analyses (Kaplan–Meier estimates and log-rank test) were conducted to examine the effect of treatment on the length of time to relapse and the length of time to lapse stratified by sex. Participants were censored if they did not experience the outcome (relapse or lapse) on or before day 84 of the trial. The primary outcome alcohol consumption variables were entered together into a MANOVA using Pillai's trace for small samples. These were the percentage of days abstinent, the number of heavy drinking days, and average drinks per drinking day at week 12. Although baseline differences in drinks per drinking day were not statistically significant, we conducted a follow-up sensitivity analysis including the covariate baseline “average drinks per drinking day” given previous reports that baseline drinking predict treatment response to baclofen (23, 31). Finally, we examined the effect of baclofen vs. placebo for each of women and men. The frequency of common adverse events (sedation and dizziness) associated with baclofen was examined between women vs. men among those participants randomized to baclofen using χ^2^ tests. All analyses were two-tailed, with a significance level at *p* < 0.05. Data were analyzed using SPSS 27 for Mac OSX.

## Results

### Patient Baseline Characteristics and Study Variables

Sociodemographic and drinking characteristics of this study sample for women and men are depicted in Table 1 as per the treatment group. There were more male than female participants consistent with the sex difference for AUD seeking treatment in Australia. There were no significant differences in baseline continuous or categorical characteristics for baclofen × sex (*p*'s > 0.08). There were no significant differences in the frequency of dose (25 vs. 75 mg) between sexes, suggesting that the dose of baclofen was distributed evenly among women and men (*p* = 0.97: women: PL = 10, 25 mg = 20, 75 mg = 10; men: PL = 23, 30 mg = 26, 25 mg = 25). There were no significant sex × treatment group differences in study completion rates (*p* = 0.81).

**Table 1:** Intention to treat: baseline characteristics of patients according to sex and treatment.

	**Placebo**		**Baclofen**	
**Characteristic**	**Women**	**Men**	**Women**	**Men**
	**(*n* = 10)**	**(*n* = 23)**	**(*n* = 20)**	**(*n* = 51)**
Age, y	48.7 ± 10.32	47.96 ± 9.96	50.10 ± 9.53	47.76 ± 10.18
Education, y	15.85 ± 2.36	13.43 ± 2.81	12.84 ± 3.46	12.97 ± 3.44
Average drinks per drinking day^a^	11.79 ± 4.19	15.15 ± 7.88	14.18 ± 7.13	16.14 ± 12.22
Years alcohol problems	18.44 ± 11.57	15.36 ± 11.70	17.4 ± 9.24	17.9 ± 12.31
Cigarette smokers, %	60	68	75	65
Lifetime Major Depression, %+	67	59	79	59
Lifetime Anxiety Disorder, %+	67	85	67	60
ADS	18.5 ± 7.53	16.91 ± 9.88	20.85 ± 8.43	19.27 ± 10.58
PACS craving	20.20 ± 4.13	16.95 ± 7.22	15.50 ± 8.67	16.47 ± 7.53
DASS Depression	15.2 ± 12.04	22 ± 12.47	17.9 ± 12.15	14.41 ± 10.63
DASS Anxiety	8.2 ± 6.9	11.64 ± 9.17	14.2 ± 7.67	12.82 ± 10.29

*Data represent mean ± SD of raw data unless otherwise noted. There were no significant differences between the groups.*

a *During the 30 days preceding the 1st day of the study, based on the Time-Line Follow-Back method. ADS, Alcohol Dependence Severity Scale; PACS, Penn Alcohol Craving Scale; DASS, Depression Anxiety Stress Scale. + as measured by the MINI Neuropsychiatric Diagnostic Interview*.

### Main Drinking Outcomes

Table 2 depicts the main drinking outcomes. At week 12, drinking data for relapse and lapse were available for 89% of subjects. Survival analyses revealed that, for women, there was a main effect of baclofen for the number of days to first lapse (Log Rank: χ^2^= 6.23, *p* = 0.01, OR: 7.74, Cohen's *d* = 0.49) but not for men (Log Rank: χ^2^= 2.48, *p* = 0.12, OR: 1.5, Cohen's *d* = 0.22) and a trend for significance for the number of days to first relapse for women (Log Rank: χ^2^ = 3.15, *p* = 0.08, OR: 4, Cohen's *d* = 0.27) but not for men (Log Rank: χ^2^ = 2.03, *p* = 0.16, OR = 2.03, Cohen's *d* = 0.17). For women, MANOVA revealed a marginal treatment effect attributed to the overall alcohol consumption outcome variables (Wilks multivariate test of significance; *F* = 2.67, *p* = 0.080) whereby further exploration observed a significant effect for percentage of days abstinent (*F* = 5.38, *p* = 0.032). Including the covariate baseline “average drinks per drinking day” reduced the treatment effect of the overall model and a significant effect for percentage of days abstinent although it remained significant (*F* = 4.61, *p* = 0.046). There were no significant overall effects of treatment for men (Wilks multivariate test of significance; *F* = 1.10, *p* = 0.36).

**Table 2:** Intention to treat: drinking outcome measures at week 12 for women and men treated with either baclofen (30–75 mg) or placebo.

**Outcome**	**Placebo**		**Baclofen**	
	**Women**	**Men**	**Women**	**Men**
	**(*n* = 10)**	**(*n* = 23)**	**(*n* = 20)**	**(*n* = 74)**
**Alcohol consumption measures**				
Time to first lapse (days) ± SEM	5.44 ± 2.66	13.52 ± 6.21	36.06 ± 8.67	24.80 ± 5.10
Time to first relapse (days) ± SEM	13.44 ± 8.61	18.05 ± 6.27	39.44 ± 8.97	31.23 ± 5.41
Percentage days abstinent	31.81 ± 12.97	48.11 ± 8.32	72.28 ± 9.17	64.36 ± 5.89
Average drinks per drinking day^+^	7.54 ± 2.42	7.07 ± 1.55	5.93 ± 1.71	5.42 ± 1.01
Number of heavy drinking days^#^^++^	1.57 ± 1.03	2.77 ± 0.66	1.79 ± 0.73	2.12 ± 0.47
**Adverse events**				
Sedation, %	n/a	n/a	45	36
Dizziness, %	n/a	n/a	20	11

*Data represent raw means ± SD unless otherwise noted. Drinks is equal to standard drink (10 g ethanol).*

# *Defined as ≥ 4 drinks for women and ≥ 5 drinks for men,*

+ *at week 12 follow-up,*

++ *per week at week 12 follow-up. AD, antidepressant, SEM, standard error of the mean. There was a significant effect of baclofen on time to lapse for women (p = 0.01) but not men (p = 0.12). There was a trend for a significant effect of baclofen for time to relapse for women (p = 0.08) but not for men (p = 0.16). There was a trend for a significant effect of baclofen for the alcohol consumption outcomes at follow-up (p = 0.08) but not for men (p = 0.36). There were no significant differences between men and women for adverse events. Exploration of dose response for adverse events revealed no significant differences (sedation for women: 10 mg = 30%, 25 mg = 60%; men: 10 mg = 18%, 75 mg = 54%; dizziness for women: 10 mg = 11%, 25 mg = 30%; men: 10 mg = 4.5%, 75 mg = 18%)*.

### Adverse Events

We explored the role of sex on the tolerability of baclofen by comparing the frequency of adverse events between women and men among those participants randomized to baclofen (see Table 2). There were no differences between groups for any adverse event (*p*'s > 0.44) whereby for sedation, women vs. men = 45% vs. 36%, and for dizziness = 20% vs. 11% respectively. The exploration of dose response for adverse events revealed no significant differences for sedation or dizziness (*p*'s > 0.61). In the 10 mg group, reported sedation in women vs. men was 30% vs. 18% respectively and was 60% vs. 54% in the 25 mg group. Reported dizziness in the 10 mg group was 11% vs. 4.5% for women versus men respectively and 30% vs. 18% in the 25 mg group.

## Discussion

The main aim of this study was to examine the role of sex in baclofen treatment response in a randomized, placebo-controlled double-blind study. Interestingly, we demonstrated that, for women, there was a main effect of baclofen for the number of days to first lapse but there was no significant effect of baclofen for men. In addition, for women, there was a marginal effect for the number of days to first relapse but not for men. We also examined other alcohol consumption outcomes (percentage of days abstinent, heavy drinking days, or average drinks per drinking day at week 12) whereby we observed a marginal effect of baclofen vs. placebo for women but not for men.

These findings are consistent with a recently completed trial by Garbutt et al. (22), which indicated the potential of an enhanced treatment effect for women compared to men. These authors found that women demonstrated a greater reduction in heavy drinking days and abstinence from alcohol (22). We similarly report that women responded more favorably to baclofen in terms of our primary outcome time to lapse and also an observed trend for our secondary outcome abstinence from alcohol. Early preclinical research demonstrated that female rodents displayed a greater reduction in cocaine self-administration than men when treated with baclofen (35). In support of these studies, it has been demonstrated that the balance of GABA_B_ presynaptic and postsynaptic activity between men and women is different (36). Moreover, electrophysiological studies have revealed differences in baseline central amygdala GABAergic spontaneous inhibitory postsynaptic currents between men and women and differences occurring across the estrous cycle stages (37). It is thus possible that some of the heterogeneities with regard to baclofen response may be due to the role of biological sex differences. Sex-specific considerations in the development of alcohol pharmacotherapy including human laboratory studies are required to examine the response across the estrous cycle and stratification of sex and reporting of female focussed data in randomized controlled trials (RCTs).

Notwithstanding, we did not observe any meaningful differences with regard to tolerability or drop out for women and men. This is inconsistent with previous reports from Garbutt et al. (22) whereby drop-outs and dose reduction from sedative side effects in high dose baclofen was 59% for women in comparison to 5% for men. While our exploratory analyses of sedative side effects for high dose (75 mg) baclofen revealed consistently higher rates in women relative to men, these were not significant (sedation was 60 vs. 54%; dizziness was 30 vs. 18% for women and men, respectively). However, the current results should be interpreted with caution given the small sample size and lack of power for these categorical outcomes in addition to limitations with measuring frequency rather than severity and that the adverse events examined were only those commonly reported across the entire sample.

To this degree, there are several limitations to this study. Firstly, we had limited power with a small sample in the placebo group for women. Most importantly, this study was not designed to directly examine the role of sex in moderating baclofen response by stratified recruitment by sex, such that baseline characteristics may not have been adequately balanced for this research question. Although there were no significant differences between sociodemographic or clinical characteristics at baseline, direct observations in larger sample sizes stratified for sex are required. As such, our sample most likely limits generalisability to other subpopulations that may have variations in baseline characteristics such as alcohol consumption.

## Conclusion

Our data provide further preliminary support for the hypothesis that baclofen may be particularly effective in women and that sex may be a potential moderator of the baclofen response in AUD. While the small sample and *post-hoc* design indicate great caution with regard to interpretation, these results highlight both the need for sex stratification in clinical trials and the development of sex-specific treatments for AUD. Longitudinal modeling of symptoms associated with AUD during treatment would further elucidate the relationship between sex and baclofen response.

## Data Availability Statement

The raw data supporting the conclusions of this article will be made available by the authors, without undue reservation.

## Ethics Statement

The studies involving human participants were reviewed and the study was approved by the Human Ethics Review Committee of the Sydney Local Health District, Northern Sydney Local Health District and South Western Sydney Local Health District (X11-0154 & X07-0041 & X01-0262) and the main trial was registered in the Clinical Trials Registry (NCT01711125). The patients/participants provided their written informed consent to participate in this study.

## Author Contributions

KM, AB, and PH contributed to study conception and design, supervision of the trial, data analysis and data interpretation, and writing of the manuscript. EL contributed to patient recruitment, data collection, and data maintenance (cleaning and checking). TH and EL contributed to manuscript preparation. PH, GD, and NP contributed as site investigators and physicians. All authors contributed to the article and approved the submitted version.

## Funding

This study was supported by a grant from the National Health and Medical Research Council of Australia, NSW Translational Research Fellowship (KM), and MRFF Next Generation Practitioner Research Fellowship (PH).

## Conflict of Interest

The authors declare that the research was conducted in the absence of any commercial or financial relationships that could be construed as a potential conflict of interest.

## Publisher's Note

All claims expressed in this article are solely those of the authors and do not necessarily represent those of their affiliated organizations, or those of the publisher, the editors and the reviewers. Any product that may be evaluated in this article, or claim that may be made by its manufacturer, is not guaranteed or endorsed by the publisher.
